# Childhood predictors of avoidant personality disorder traits in adolescence: a seven‐wave birth cohort study

**DOI:** 10.1111/jcpp.14064

**Published:** 2024-11-04

**Authors:** Lars Wichstrøm, Theresa Wilberg, Elfrida Hartveit Kvarstein, Silje Steinsbekk

**Affiliations:** ^1^ Department of Psychology Norwegian University of Science and Technology Trondheim Norway; ^2^ Department of Child and Adolescent Psychiatry St Olavs Hospital Trondheim Norway; ^3^ Division of Mental Health and Addiction Department for Research and Innovation Oslo Norway; ^4^ Institute for Clinical Medicine University of Oslo Oslo Norway; ^5^ Section for Personality Psychiatry and Specialized Treatments, Division of Mental Health and Addiction, Department for National and Regional Functions Oslo University Hospital Oslo Norway

**Keywords:** Adolescent, attachment, avoidant personality disorder, childhood, longitudinal

## Abstract

**Background:**

Although it is widely assumed that avoidant personality disorder (AvPD) originates in childhood, there is little prospective research to substantiate this claim. We therefore aimed to determine whether presumed childhood risk factors predict AvPD traits at 16 years.

**Methods:**

A population‐based sample (*n* = 1,077; 50.9% female) from the 2003 and 2004 birth cohorts in Trondheim, Norway was examined biennially from 4 to 16 years. The number of AvPD traits at the age of 16 was assessed with the structured clinical interview for DSM‐5 personality disorders and regressed on the intercept and growth in child risk and protective factors until the age of 14.

**Results:**

The prevalence of AvPD at the age of 16 was 3.2% (95% CI: 2.2–4.1). Higher levels and an increased number of social anxiety symptoms over time, as well as increased negative affectivity/neuroticism, predicted a higher number of AvPD traits. When the levels and changes in these factors were adjusted for, less and decreasing extraversion forecasted more AvPD traits, as did declining self‐worth, higher levels of parental AvPD traits, and increased onlooking behavior.

**Conclusions:**

Neuroticism, low extraversion, social anxiety symptoms, passive onlooking behavior, and low self‐worth predicted a higher number of AvPD traits in adolescence, as did more AvPD traits in parents. Efforts to enhance self‐worth, reduce social anxiety, and promote peer interaction among onlooking children may reduce the development of AvPD traits in adolescence.

## Introduction

Avoidant personality disorder (AvPD) is characterized by a pervasive pattern of avoiding social interaction, hypersensitivity to negative evaluations from others, and feelings of social inadequacy. It is among the most prevalent personality disorders (PDs) and affects 2.7% of the population (Winsper et al., 2020). The prevalence in clinical populations ranges from 11% to 57% (Hummelen, Wilberg, Pedersen, & Karterud, 2006). Adults with AvPD experience a variety of functional incapacities, including reduced somatic health, solitariness, unemployment, and low educational attainment (Kvarstein, Antonsen, Klungsoyr, Pedersen, & Wilberg, 2021; Skodol et al., 2002). To aid planning and treatment efforts, knowledge about the etiology of AvPD is essential, which longitudinal studies may inform.

### Potential predictors

At present, there is no overarching theoretical model for the development of AvPD. Even so, most scholars consider a range of child factors to constitute an early emerging vulnerability for reacting with fear of rejection, leading to withdrawal from challenging social interaction. Such behavior may have cascading effects by increasing interaction difficulties, fueled by increased vulnerability (Lampe & Malhi, 2018). Hence, it is important to consider both child and social factors when identifying potential etiological contributors.

#### Child factors

From a vulnerability perspective, the Diagnostic and Statistical Manual of Mental Disorders fifth edition (DSM‐5) (American Psychiatric Association, 2013) argues that AvPD starts in childhood with shyness, isolation, and fear of new people and situations. Shyness and fear are considered the social and non‐social aspects, respectively, of behavioral inhibition (Wichstrøm, Belsky, & Berg‐Nielsen, 2013). Both are narrow constructs belonging to the overarching temperamental/personality dimensions of surgency/extraversion (i.e. low extraversion; introversion) and negative affectivity/neuroticism, respectively, which are strongly correlated with AvPD traits (Welander‐Vatn et al., 2019). Thus, the question arises whether AvPD is first and foremost linked to the narrow constructs of fear and shyness or the broader temperamental and personality dimensions. Moreover, as feelings of inferiority are a trait of AvPD, one should also expect low self‐esteem to be a forerunner of AvPD. Arguably, the fear of rejection seen in AvPD may be, in part, rooted in doubt that one has the skills to deal with situations that might result in rejection or ridicule. Thus, self‐assertiveness should be particularly impeded. It has also been speculated that problems with mentalizing, that is, reading the inner states of others, may contribute to the social interaction problems seen in AvPD (Moroni et al., 2016). Moreover, such mentalizing difficulties are strongly linked to reduced affect consciousness in patients with AvPD (Johansen et al., 2018). Thus, limited development of mentalizing others' *emotions*, that is, the normative growth and refinement of emotion understanding seen through childhood (Pons, Harris, & de Rosnay, 2004), might serve as a vulnerability for developing AvPD. Others have drawn attention to the possibility that more general cognitive skills may be impaired in AvPD, especially cognitive flexibility—a core element of executive functions. When facing the possibility of rejection, some children may have limited ability to think and behave differently (Eggum et al., 2009), potentially causing social difficulties and withdrawal. Impaired executive functions may thus be a precursor of AvPD.

Unsurprisingly, AvPD is strongly related to social anxiety disorder (SAD), and questions have been raised about whether AvPD is an extreme and pervasive form of SAD (Chambless, Fydrich, & Rodebaugh, 2008). However, more recent studies find the symptomatology, genetics, and environmental contributions to only partly overlap (Lampe & Malhi, 2018; Torvik et al., 2016; Welander‐Vatn et al., 2019), warranting separate constructs. Even so, there are reasons to expect SAD to be a precursor to AvPD, either by contributing to it (e.g. through starting a circle of fear of rejection and withdrawal) or by shared risk factors.

Further, numerous studies find adult patients with AvPD to be characterized by an anxious/avoidant attachment style (Lampe & Malhi, 2018), and, accordingly, it is a widespread assumption that avoidant attachment is part of the etiology of AvPD (Lampe, 2016). Such an attachment style is characterized by internal working models that prescribe that others cannot be trusted and that one is ineffective in social relations, leading to a need for emotional and social detachment and self‐reliance (George & Solomon, 1989).

Finally, scholars have speculated that the above personal vulnerabilities might increase the risk of developing a pattern of ‘onlooking behavior,’ which involves wanting to affiliate with peers but also being afraid to join in—a type of approach–avoidance conflict, that could grow into AvPD (Sorensen, Wilberg, Berthelsen, & Rabu, 2020).

#### Social factors

Several of the above personal vulnerabilities likely have social interactional origins, at least in part [e.g. attachment (George & Solomon, 1989); emotion understanding (Laugen, Kårstad, Reinfjell, & Wichstrom, 2024); executive functions (Halse, Steinsbekk, Hammar, Belsky, & Wichstrøm, 2019); and social anxiety (Ollendick & Benoit, 2012)]. Even so, distinct environmental factors, possibly intertwined with the above child factors, have been suggested to contribute to AvPD. These include adverse childhood experiences (ACEs) (Lampe & Malhi, 2018), bullying victimization or peer rejection (Sorensen et al., 2020), and having few positive relationships with adults (Rettew et al., 2003). Moreover, given the heritability of AvPD and the potentially pathogenic parenting styles among adults struggling with avoidant traits (Steele, Townsend, & Grenyer, 2019; Wichstrøm, Borgen, & Steinsbekk, 2023), AvPD traits in parents should be considered.

### The need for prospective research

Except for links between officially recorded abuse and neglect (Cohen, Crawford, Johnson, & Kasen, 2005), the above suggestions have been based on retrospective reports from adults—often patients with AvPD. Retrospective reports of presumed formative childhood factors, such as maltreatment, neglect, and abuse (Baldwin, Reuben, Newbury, & Danese, 2019), and the quality of parenting (Nivison, Vandell, Booth‐LaForce, & Roisman, 2021) evince little overlap with prospective reports from childhood, and an individual's current mood may color retrospective reports (Dalgleish & Werner‐Seidler, 2014). Consequently, the ability of available findings to help elucidate the etiological mechanism involved in AvPD is limited. No prospective studies—except for the Children in Community Study from 1975 (Cohen et al., 2005)—exist; hence, prospective research starting in childhood is needed.

### The development of predictors

Although knowledge about risk factors in the first years of life may be informative, the continuity in assumed predictors is only modest to moderate throughout childhood, even those alleged to be substantially constitutional, such as temperament and personality traits (Kopala‐Sibley, Olino, Durbin, Dyson, & Klein, 2018). Therefore, many children with presumed high early risk of AvPD may have a more positive developmental course, and the risk of AvPD in these children should thereby be expected to subside compared to those whose risk exposure or risk traits continue or aggravate. Because of these differential developmental trajectories, we argue that not only the *level* of risk at a single time point but also the *development* of risk throughout childhood should be considered.

### Current study

Although the DSM‐5 conceptualizes PDs as categorical, it also acknowledges that current research supports a dimensional view. For this and power reasons, we used a dimensional approach. Moreover, even though PD diagnoses are typically not set before adulthood, there is much evidence indicating that PD traits can also be validly identified in adolescence (d'Huart et al., 2023). In sum, we hypothesized that (i) the initial levels (intercept) in the preschool or early school years and (ii) changes (growth) in suggested risks and protectors through middle childhood and early adolescence would predict AvPD traits at 16 years of age. We focused on personal factors (i.e. female sex, neuroticism, extraversion, temperamental fear and shyness in childhood, SAD symptoms, avoidant attachment, onlooking behavior, emotion understanding, assertiveness, and executive functioning) and external factors (i.e. parental AvPD traits, serious negative life events, bullying victimization, and closeness in the student–teacher relationship), exploiting a birth cohort sample assessed biennially from 4 to 16 years of age.

## Methods

### Procedure and participants

A letter inviting the 2003–2004 birth cohorts and their parents in the city of Trondheim, Norway (*N* = 3,456) to participate in the Trondheim Early Secure Study (TESS) (Steinsbekk & Wichstrøm, 2018)was enclosed with an invitation to a regularly scheduled community health screening for 4‐year‐olds. The letter also contained the Strengths and Difficulties Questionnaire (SDQ) (Goodman, Ford, Simmons, Gatward, & Meltzer, 2000), a screening tool for emotional and behavioral problems in children. Of the 3,358 parents presenting to the well‐child clinic, 2,477 consented to participate (176 were excluded due to inadequate language proficiency, and 166 were missed due to not being asked by the health nurse). To enhance variability and, thus, power, we oversampled for children with behavioral and emotional problems. To this end, children were divided into four strata according to their SDQ score (cutoff points 0–4, 5–8, 9–11, and 12–40). Children with higher scores had a higher probability of being selected for the study (.37, .48, .70, and .89 in each stratum). This oversampling was corrected for in the analyses. In all, 1,250 families were drawn into the study; we obtained information from 1,007 families at the first wave (T1; *M*_age_ = 4.7, *SD* = 0.3; 50.9% girls). The participants were reassessed biennially until the age of 16 (Figure S1). Little's MCAR test (Little, 1988) indicated that data were missing completely at random (χ^2^ = 84,209, *df* = 88,780, *p =* 1.00). Demographic information is provided in Table 1. Most children were of Norwegian origin; 84.8% of their parents were married or were cohabitating for more than 6 months; 63.9% of the mothers and 51.9% of the fathers had at least a bachelor's degree. *The project was approved by* the Regional Committee for Medical and Health Research Ethics, Mid‐Norway (approval number 2019/509). Health nurses informed parents about the study, and written consent to participate was obtained from parents. At 16 years of age, the legal consent age in Norway for medical and research purposes, written consent was obtained from the adolescents themselves.

**Table 1:** Sample characteristics (*n* = 1,007)

Demographic characteristics	*N*	%
Gender of child		
Boy	510	51.6.
Girl	497	49.4
Biological parents' marital status		
Married	538	53.4
Cohabitating >6 months	317	31.4
Separated	20	2.0
Divorced	77	7.6
Widowed	2	0.2
Cohabitating <6 months	13	1.3
Never lived together	16	1.6
Missing information	23	2.3
Ethnic origin of biological mother		
Norwegian	911	90.4
Western countries (Western Europe, USA, Australia, New Zealand)	32	3.2
Other countries	42	4.1
Missing information	23	2.3
Ethnic origin of biological father		
Norwegian	889	88.2
Western countries (Western Europe, USA, Australia, New Zealand)	61	6.1
Other countries	33	3.2
Missing information	25	2.5
Mother's highest completed education		
Junior high school not completed	0	0
Junior high school (10th grade)	6	0.6
Some education after junior high school	60	6.0
Senior high school (13th grade)	169	16.8
Some education after senior high school	35	3.5
Some college or university education	78	7.8
Bachelor's degree	62	6.2
College degree (3–4 years of study)	336	33.4
Master's degree or similar	202	20.0
PhD, ongoing	12	1.2
PhD, completed	31	3.1
Missing information	17	1.7
Father's highest completed education		
Junior high school not completed	3	0.3
Junior high school (10th grade)	27	2.7
Some education after junior high school	52	5.2
Senior high school (13th grade)	175	17.4
Some education after senior high school	42	4.2
Some college or university education	64	6.3
Bachelor's degree	33	3.3
College degree (3–4 years of study)	214	21.2
Master's degree or similar	208	20.6
PhD, ongoing	15	1.5
PhD, completed	53	5.3
Missing information	122	12.1

### Measures

#### Avoidant personality disorder traits

AvPD traits and the diagnosis of AvPD were measured at 16 years with the Structured Clinical Interview for DSM‐5 Personality Disorders (SCID‐5‐PD) (First, Williams, Benjamin, & Spitzer, 2016). In the SCID‐5‐PF, a distinction is made between absent (score 0), below threshold (scored 1), and at or above threshold (score 2). A score of 2 is assigned when the trait meets the specific criteria for this trait and the general criteria for a PD. A score of 1 is used when the inner experiences or behavior of the person are present but not to the extent that meets the diagnostic criteria of severity, duration, impairment, and pervasiveness. A sum score of traits (coded either 0, 1, or 2) was created. Trained interviewers had at least a BA in relevant fields and extensive experience interviewing adolescents with clinical psychiatric interviews. The interviews were audiotaped, and 114 tapes were recoded by blinded raters, yielding an interrater reliability (ICC) of 0.84.

#### Neuroticism and extraversion

Temperament could be considered the childhood constitutional foundation from which personality traits develop (Rothbart, Ahadi, & Evans, 2000). There are strong empirical continuities between the temperament and personality traits we investigated here—negative affectivity (i.e. discomfort, frustration, sadness, fear, and being difficult to soothe) and neuroticism and surgency (i.e. high activity levels, high‐intensity and pleasure‐seeking, low shyness, and impulsivity) and extraversion (Shiner & Caspi, 2003). We, therefore, examined the growth in neuroticism and extraversion with negative affectivity and surgency, respectively, included as the early manifestations of these personality traits. At 4 and 6 years of age, negative affectivity (α = .77 and .81) and surgency (α = .77 and .83) were measured by the short form of the Children's Behavior Questionnaire (Rothbart, Ahadi, Hershey, & Fisher, 2001), which was completed by parents. At 10, 12, and 14 years of age, the Big Five Inventory (Soto, John, Gosling, & Potter, 2008), completed by the children, was applied to measure neuroticism (α = .59, .72, .81, respectively) and extraversion (α = .54, .67, .75, respectively).

#### Temperamental fear and shyness

The negative affectivity and surgency dimensions of the Children's Behavior Questionnaire (above), measured at ages 4 and 6, include the subscales of fear and shyness, respectively. The fear scale captures anxiety related to anticipated pain or distress and/or potentially threatening situations (α = .73 and .72 at ages 4 and 6, respectively), whereas the shyness scale measures a slow or inhibited approach to novel situations or new people (α = .90 and .80, respectively).

#### Symptoms of social anxiety disorder

We applied the Child and Adolescent Psychiatric Assessment (CAPA; Angold & Costello, 2000) to capture symptoms of SAD. The children and their parents were interviewed separately. The CAPA contains mandatory questions and optional follow‐ups, and interviewers continue to probe until a decision can be made regarding whether a symptom is present, either reported by the child or the parent. A 3‐month primary period was employed, and symptom onset, duration, and intensity were recorded. A symptom count (range = 0–2) was applied. Blinded coders recoded 77 CAPA interviews conducted at 10 years of age, yielding an interrater reliability (ICC) of 0.88.

#### Onlooking behavior

At 6–14 years, the teacher who knew the child best completed the Conflicted shyness subscale of the Child Social Preference Scale (Coplan, Prakash, O'Neil, & Armer, 2004). The scale was created to tap approach–avoidance conflict in peer relations by items such as ‘The child seems to want to play with others but is sometimes nervous too,’ rated on a 5‐point Likert scale (α = .82–.89).

#### Emotion understanding

The Test of Emotion Comprehension (TEC) (Pons et al., 2004) was included at 4, 6, and 8 years of age. The TEC is administered in booklet form, with each page displaying a drawing of a child in a situation, for example, receiving a present. The face of the protagonist is left blank, and four alternative facial expressions representing different emotions are displayed at the bottom of the page. The experimenter reads a vignette about the scene and asks the child to point at the facial expression that might represent the character's emotions. The TEC contains 21 items that are the same for children of all ages and are divided into nine components. One point is given for each component the child masters. Because all components are scored dichotomously, Armor's theta (Armor, 1973) was used as a measure of reliability (θ = .82–.86).

#### Executive functioning

We applied the Behavior Rating Inventory of Executive Functions (BRIEF) (Gioia, Isquith, Guy, & Kenworthy, 2000), which was completed by the teacher who knew the child best at 6 to 14 years of age. The BRIEF captures eight aspects of executive functioning: inhibition, cognitive flexibility, working memory, planning/organizing, emotional control, initiation, organization of materials, and monitoring (Gioia, Kenworthy, & Isquith, 2010). The BRIEF is reported to have satisfactory internal consistency and convergent validity (Ezpeleta, Granero, Penelo, de la Osa, & Domenech, 2015). In the present inquiry, we used the total scale score (α = .98 at all ages).

#### Assertiveness

The Assertiveness dimension of the Social Skills Rating System (SSRS) (Gresham & Elliott, 1990) was applied. At 4 years of age, the preschool version was used (10 items; α = .81); at 6, 8, and 10 years of age, we applied the elementary version (10 items; α = .81 at all ages). The Social Skills Improvement System (SSiS) (Gresham & Elliott, 2007), which is a further development of the SSRS, was used at 12 and 14 years of age to assess assertiveness (seven items; α = .76 and .77 at 12 and 14 years of age, respectively). Parents rated all items of the Norwegian versions on a 4‐point scale (1 = ‘never’ to 4 = ‘very often’).

#### Self‐worth

To assess global self‐worth, the Self‐Description Questionnaire I (SDQ‐I) (Marsh, Barnes, Cairns, & Tidman, 1984) was administered to children at 6, 8, and 10 years of age, containing eight items that are rated on a 5‐point Likert scale (1 = *wrong* to 5 = *true*; α = .82–.87). At 12 and 14 years of age, the corresponding age‐appropriate five‐item Global Self‐Worth Scale of the Revised Self‐Perception Profile for Adolescents (SPPA‐R; α = .79–.84) was applied (Harter, 1988; Wichstrøm, 1995) using a 4‐point scale ranging from 1 = ‘describes me very poorly’ to 4 = ‘describes me very well.’

### Avoidant attachment

#### Ages 4 and 6: The Manchester Child Attachment Story Task (MCAST)

The children's attachment representations were assessed at ages 4 and 6 using the MCAST. The MCAST is suitable for children aged 4–8 (Green, Stanley, Smith, & Goldwyn, 2000) and is set up as a doll‐play completion task involving one doll representing the child and one doll representing the parent who accompanied the child to the assessment. The administrator establishes a story that includes a child doll and a mummy or daddy doll.

The MCAST stories begin with everyday/neutral events followed by a sudden and distressing event where the child: (a) is alone when waking up from a nightmare in the middle of the night, (b) hurts a knee while biking (close to their home), having pain and bleeding, (c) experiences acute abdominal pain when watching TV alone in the living room (with parent present in the kitchen nearby), and (d) becomes lost while with the parent at a large shopping mall. This format is designed to activate the child's attachment system and, hence, attachment‐related behaviors and thoughts, which resemble those used in the Strange Situation Procedure and the ‘five adjective questions’ in the Adult Attachment Interview (AAI; George, Kaplan & Main, 1996; Main & Goldwyn, 1994). As the story climaxes, the administrator asks, ‘What happens next?’ to facilitate the completion of the child's narrative. The child is then asked about the feelings experienced by the child and the parent doll.

The child's narrative and behavior in each vignette are assigned a primary attachment strategy (A, B, C, or D) and a secondary strategy if relevant. Following the procedure described in Viddal et al., a mean score of avoidant attachment across the four vignettes was created (Viddal, Berg‐Nielsen, Belsky, & Wichstrom, 2017). The MCAST creators trained administrators and coders. The entire MCAST procedure was videotaped, and from T1 and T2, 157 videos were randomly drawn and recoded by blinded coders (ICC = 0.71).

#### Ages 10, 12, and 14: Middle Childhood Attachment Strategies Coding System (MCAS)

Following the MCAS procedure (Brumariu et al., 2018), the participating child and one parent were observed and videotaped during three interaction tasks. First, the parent and the child were asked to play a cooperative game for 3 min. Second, they were asked to identify and agree on the most important area on which they disagreed and then discuss it for 8 min. The dyads were free to choose a topic on their own but were provided with an age‐appropriate list of topics that parents and children often disagree on (e.g. chores, bedtimes, screen use, homework, and clothing).

Organized around the construct of a secure base, the MCAS assesses behavioral manifestations of the four attachment strategies identified in infancy (i.e. secure, avoidant, ambivalent, and disorganized‐disoriented). A 9‐point rating scale is used for each of the six attachment styles, ranging from 1 = none or slight/isolated minor evidence of a specific pattern that does not characterize the child's behavior and interaction overall to 9 = marked and persistent evidence of a specific pattern that predominantly characterizes the child's behavior and interaction. Coders were trained and certified by the originators of the MCAS and were unaware of any information about the family. From T4 to T6, 315 videos were randomly drawn and recoded by blinded raters (ICC = 0.66).

### Parental avoidant personality disorder traits

The DSM‐IV and ICD‐10 Personality Questionnaire (Ottosson et al., 1998) is a self‐report questionnaire used to capture DSM‐IV and ICD‐10 PDs. The AvPD scale consists of seven items rated dichotomously as ‘true’ or ‘false.’ Favorable reliability and validity in clinical as well as nonclinical samples have been documented (Ottosson et al., 1995, 1998; Ottosson, Grann, & Kullgren, 2000). The measure was administered to the parent accompanying the child at 4, 6, and 14 years of age, and the internal consistencies were θ = .87, .89, and .94, respectively.

### Closeness in the student–teacher relationship

The 11‐item Closeness subscale of the student–teacher relationship scale (Pianta, 2001) was used to measure teacher‐perceived closeness in her or his relationship with the child at 4–14 years of age. The child's primary teacher completed the scale, which uses a 5‐point Likert scale ranging from 1 = *definitely does not apply* to 5 = *definitely applies* (α = .68–.85).

### Bullying victimization

The child's primary teacher completed the five‐item Olweus Bully Victim Questionnaire (OBVQ) (Solberg & Olweus, 2003) at 6 to 14 years of age to record direct and indirect forms of bullying during the previous 3 months. To capture the children's perceptions of being bullied, an eight‐item version of the OBVQ was applied, also including cyberbullying. Items are rated on a 5‐point Likert scale (1 = *never*, 2 = *rarely*, 3 = *1–3 times a month*, 4 = *1–4 times a week*, and 5 = *every day*), and midpoint values were used to represent the number of days per month (α = .66–.79 for the teacher version and α = .76–.84 for the child version).

### Serious negative life events

Parents and children (from age 8) reported whether the child had experienced any of 26 events, including the death of a loved adult, a serious vehicular accident, being burned, near‐drowning, a severe fall, witnessing violence or death, or physical and/or sexual abuse during the preceding 2 years. An event was considered present if reported by either the child or parent. A summed score of events was determined.

## Analysis plan

The development of the predictors in this inquiry throughout childhood has not always been delineated in prior research, but we expected that their growth would not necessarily be linear. Nonlinearity may also appear because instruments must be changed to include age‐appropriate measures of the predictors (i.e. attachment, temperament/personality, assertiveness, self‐esteem). To accommodate such deviations from linearity with an unknown form, we applied a latent basis growth model (Grimm, Ram, & Hamagami, 2011) in Mplus 8.5 where the shape of the growth was estimated from the data by anchoring the growth at the start of the measurement of the predictor, where the intercept was set, and the last measurement of the predictor. The growth was parameterized as year change. The number of AvPD traits was regressed on the two growth factors, intercept (level) and slope (change), which were allowed to correlate. We conducted univariate regressions first and thereafter adjusted for sex and the intercepts and growth in the two most well‐documented predictors: neuroticism and SAD symptoms. As symptoms of SAD are rare before the age of 10 (Steinsbekk, Ranum, & Wichstrøm, 2022), we included the linear level and slope in such symptoms from 10 to 14 years of age. To adjust for oversampling, population weights were applied, corresponding to the number of children in the population in a stratum divided by the number of children drawn to participate from that stratum using a sandwich estimator. A robust maximum likelihood estimator was employed, providing robust standard errors, and missing data were handled through a full information maximum likelihood procedure, using all available data.

## Results

### Prevalence

At 16 years of age, 3.19% (95% CI: 2.24–4.07) of the children fulfilled the diagnostic criteria for AvPD, and the mean number of traits in the population was 0.77 (CI 0.63, 0.91), with 64.82% of the children having no traits. The frequency of each trait is depicted in Table 2. A confirmatory factor analysis treating AvPD traits as ordered categorical variables revealed that a one‐factor solution fit the data well (χ^2^ (14) = 10.71, *p* = 1.000, RMSEA = .000 (90% CI: 0.000–0.029), CFI = 1.000, TLI = 1.000), with standardized factor loadings ranging between 0.66 and 0.90. The latent basis growth models of the predictors generally fit the data well (Table 3).

**Table 2:** Estimated prevalence of AvPD traits in the population below and above the diagnostic threshold (*n* = 647)

Traits	Below threshold		Above threshold	
	Prevalence % (*n*)	95% CI	Prevalence % (*n*)	95% CI
Avoids occupational activities that involve significant interpersonal contact because of fears of criticism, disapproval, or rejection	5.86 (38)	4.61–7.41	3.40 (22)	2.46–4.68
Is unwilling to get involved with people unless certain of being liked	6.29 (41)	4.97–7.93	0.85 (6)	0.46–1.57
Shows restraint within intimate relationships because of the fear of being shamed or ridiculed	6.11 (39)	4.82–7.70	1.79 (12)	1.12–2.84
Is preoccupied with being criticized or rejected in social situations	10.74 (69)	8.94–12.84	2.77 (18)	1.92–3.99
Is inhibited in new interpersonal situations because of feelings of inadequacy	8.06 (52)	6.57–9.85	2.30 (15)	1.59–3.31
Views self as socially inept, personally unappealing, or inferior to others	6.20 (40)	4.89–7.82	2.23 (14)	1.52–3.26
Is unusually reluctant to take personal risks or to engage in any new activities because they may prove embarrassing	6.47 (42)	5.14–8.11	1.34 (9)	0.80–2.24

AvpD, avoidant personality disorder.

### Predictions

#### Child factors

Girls had more AvPD traits than boys, but this difference disappeared once the levels and changes in neuroticism and social anxiety were adjusted for (Table 4). Indeed, children who scored higher on negative affect/neuroticism and social anxiety and those who showed increases in these respects throughout childhood evinced more AvPD traits. Higher, as well as increasing levels of surgency/extraversion, predicted a reduced number of AvPD traits, even when sex, neuroticism, and social anxiety were adjusted for. The above temperamental traits of negative affectivity and surgency contain the two traits mentioned explicitly in the DSM‐5 as forerunners of AvPD: fear and shyness, respectively. In univariable models, the intercepts in fear (*B* = 0.10, 95% CI: −0.08 to 0.27) and shyness (*B* = 0.09, 95% CI: −0.17 to 0.34) and changes in fear (*B* = 0.24, 95% CI: −0.25 to 0.72) and shyness (*B* = 0.22, 95% CI: −0.23 to 0.66) from 4 to 6 years of age did not predict AvPD traits. As portrayed in Table 4, higher and increasing onlooking behavior forecasted more AvPD traits, but the prediction by increasing onlooking behavior disappeared when sex, neuroticism, and SAD symptoms were adjusted for, whereas the prediction from the level of onlooking behavior remained. Lower (intercept) and decreasing (slope) self‐worth also forecasted more AvPD traits, and the prediction by diminishing self‐worth remained in the multivariable analysis. Avoidant attachment, emotion understanding, executive functioning, and assertiveness did not predict AvPD traits.

**Table 3:** Descriptives of the growth parameters of predictors of AvPD traits

Predictor (age range in years)	*n*	Growth parameters			Model fit				
		Intercept	Slope	*p*‐value of Slope	χ^2^	*df*	*p*‐value	CFI	RMSEA (90% CI)
Child factors									
Neuroticism (4–14)	957	3.64	−0.12	<.001	21.50	7	.001	.974	.052 (0.029 to 0.077)
Symptoms of social anxiety disorder^a^ (10–14)	725	0.03	0.01	<.001	0.57	1	.45	1.00	.00 (0.000 to 0.089)
Avoidant attachment (4–14)	953	0.21	0.22	<.001	5.51	7	.598	1.00	.00 (0.000 to 0.034)
Extraversion (4–14)	957	4.54	−0.09	<.001	36.23	7	<.001	.968	.066 (0.046 to 0.088)
Emotion understanding^a^ (4–8)	976	3.36	1.05	<.001	.000	0	.000	1.000	.000 (0.000 to 0.000)
Executive functioning problems^b^ (6–14)	851	88.28	−0.11	.33	18.08	6	.006	.971	.049 (0.024 to 0.075)
Onlooking behavior (6–14)	851	9.97	0.19	<.001	24.43	7	.001	.932	.054 (0.032 to 0.078)
Assertiveness (6–14)	991	2.85	0.03	<.001	61.87	7	<.001	.951	.068 (0.052 to 0.085)
Self‐worth (6–14)	808	5.02	0.01	<.001	12.11	7	.096	.977	.030 (0.000 to 0.058)
Social factors									
Parent symptoms of avoidant personality disorder (4–14)	953	1.01	0.01	.73	0.11	1	.74	1.000	.000 (0.000 to 0.060)
Closeness in student–teacher relationship (4–14)	1,002	41.32	−0.78	<.001	55.42	12	<.001	.900	.067 (0.051 to 0.085)
Serious negative life events (4–14)	1,068	0.73	−0.03	<.001	18.30	12	.076	.948	.025 (0.000 to 0.045)
Bullying victimization, teacher‐reported (6–14)	850	4.57	−0.24	.001	8.29	7	.31	.941	.015 (0.000 to 0.046)
Bullying victimization, self‐reported (8–14)	746	11.36	−1.22	<.001	1.24	3	.74	1.000	.000 (0.000 to 0.043)

Latent basis growth models.

AvpD, avoidant personality disorder; CFI, comparative fit index; RMSEA, root mean square error of approximation.

^a^ Linear growth model.

^b^ Executive functioning at ages 10 and 12 years was correlated.

Concerning social factors, a higher number of parental AvPD traits predicted more AvPD traits in children, and children exposed to more negative life events also had more AvPD traits. The latter prediction, however, disappeared in the multivariable analysis when sex, neuroticism, and SAD symptoms were included in the model. Bullying victimization and closeness in the student–teacher relationship were unrelated to later AvPD traits.

**Table 4:** Intercept and growth of childhood factors predicting the number of avoidant personality disorder traits at 16 years of age

Predictor (age range in years)	Univariable analysis				Multivariable analysis^a^			
	Intercept		Growth		Intercept		Growth	
	*B* (95% CI)	*p*‐value	*B* (95% CI)	*p*‐value	*B* (95% CI)	*p*‐value	*B* (95% CI)	*p*‐value
Child factors								
Female sex	0.46 (0.19 to 0.74)	<.001	NA		0.19 (−0.10 to 0.49)	.09	NA	
Neuroticism (4–14)	0.53 (0.33 to 0.74)	<.001	5.27 (3.74 to 6.80)	<.001	0.75 (−0.03 to 1.52)	.058	7.95 (2.22 to 13.69)	.007
Symptoms of social anxiety disorder (10–14)	1.25 (0.62 to 1.89)	<.001	5.39 (1.40 to 9.37)	.008	2.70 (0.48 to 4.93)	.017	14.07 (2.25 to 25.90)	.020
Avoidant attachment (4–14)	−4.15 (−14.77 to 5.75)	.39	0.94 (−2.82 to 4.70)	.63	−1.82 (−9.91 to 6.28)	.66	0.38 (−2.38 to 3.14)	.79
Extraversion (4–14)	−1.46 (−1.95 to −0.97)	<.001	−14.24 (−19.13 to −9.35)	<.001	−0.64 (−1.18 to −0.09)	.022	−9.27 (−17.32 to −1.22)	.024
Emotion understanding (4–8)	−0.09 (−0.44 to 0.26)	.61	0.54 (−0.87 to 1.94)	.45	−0.094 (−0.83 to 0.65)	.81	2.13 (−5.63 to 9.89)	.59
Executive functioning problems (6–14)	0.01 (−.003 to 0.024)	.13	0.23 (−0.04 to 0.51)	.09	−.02 (−0.13 to 0.09)	.74	0.95 (−2.03 to 3.93)	.57
Onlooking behavior (6–14)	0.18 (0.10 to 0.27)	<.001	1.28 (0.29 to 2.27)	<.001	0.10 (0.01 to 0.18)	.028	0.85 (−0.07 to 1.77)	.057
Assertiveness (6–14)	−0.49 (−1.30 to 0.32)	.23	−14.50 (−45.83 to 16.3)	.36	−0.38 (−1.29 to 0.52)	.41	−25.44 (−65.26 to 14.38)	.133
Self‐worth (6–14)	−0.82 (−1.13 to −0.50)	<.001	−6.24 (−11.03 to −1.45)	.003	0.09 (0.03 to 0.16)	.76	−.03 (−0.05 to −.02)	.005
Social factors								
Parent symptoms of avoidant personality disorder (4–14)	0.30 (0.11 to 0.48)	.002	−0.37 (−2.87 to 2.12)	.85	0.35 (0.12 to 0.55)	.002	−2.95 (−13.32 to 7.42)	.57
Closeness in student–teacher relationship (4–14)	−0.25 (−0.98 to 0.47)	.49	−0.11 (−0.90 to 0.68)	.78	−0.02 (−0.07 to 0.04)	.55	−0.73 (−3.26 to 1.80)	.57
Serious negative life events (4–14)	1.84 (0.15 to 3.53)	.03	10.17 (−5.19 to 25.54)	.19	−0.07 (−0.17 to 0.02)	.13	−3.15 (−26.84 to 20.54)	.79
Bullying victimization, teacher‐reported (6–14)	0.50 (0.42, 0.58)	.42	0.29 (0.15, 0.44)	.64	−0.03 (−0.38, 0.33)	.89	0.01 (−0.04 to 0.07)	.60
Bullying victimization, self‐reported (6–14)	0.37 (−0.19, 0.94)	.19	−0.45 (−2.60, 1.70)	.68	0.88 (−6.93, 8.69)	.82	−0.04 (−0.13 to 0.04)	.33

NA, not applicable.

^a^ Adjusted for sex, neuroticism, and symptoms of social anxiety disorder.

## Discussion

Although it is widely assumed that AvPD originates in childhood, there is little prospective research to substantiate this claim. Therefore, we examined whether the level and growth in a comprehensive set of presumed childhood risk factors predicted the number of AvPD traits at 16 years of age. We did so by following a birth cohort sample with biennial assessments from 4 years of age. Several risk factors identified by previous retrospective or concurrent research on adults with AvPD received support: higher—and increasing—neuroticism and SAD symptoms predicted more AvPD traits. Over and above these effects, higher—and increasing—extraversion forecasted fewer AvPD traits, and children with declining self‐worth, more onlooking behavior, and parents who had high levels of AvPD traits were at increased risk. This being said, many presumed risk factors failed to emerge as predictors (i.e. temperamental fear and shyness, avoidant attachment, reduced understanding of others' emotions, being a victim of bullying, lack of closeness in the primary student–teacher relationship, low assertiveness, and serious negative life events) or failed to do so once sex, neuroticism, and the number of social anxiety symptoms were adjusted for.

The DSM‐5 highlights two traits, fearfulness and shyness, to start the development of AvPD. This view was not supported by the data, as neither fear nor shyness turned out to be predictive in the current inquiry. Instead, the broader constructs of neuroticism and (reduced) extraversion (i.e. introversion), which subsume fearfulness and shyness, respectively, forecasted AvPD traits. Hence, the beginning of a developmental process toward AvPD may not necessarily start with the specific traits that resemble AvPD the most—shyness and fear—but rather with the broader temperamental traits of negative affectivity and low surgency.

Given that AvPD is highly comorbid with SAD and may share many of the same risk factors, it has been proposed that AvPD is a persistent and severe form of SAD (Reich, 2000). However, more recent research has found that SAD and AvPD traits are differently related to Big Five personality traits (Welander‐Vatn et al., 2019), and differential concurrent relations to social cognition, self‐concept, and genetics have been indicated (Lampe & Malhi, 2018). Our results are consistent with the latter view in that several childhood factors predicted AvPD traits even when the number of SAD symptoms was adjusted for. We extend previous research by showing that decreasing self‐worth, more onlooking behavior, neuroticism, and low extraversion in childhood are prospectively predictive of later AvPD traits over and above social anxiety.

The identification of AvPD traits in parents to predict AvPD traits in their offspring is consistent not only with the high heritability of AvPD (Reich & Schatzberg, 2021) but also with the view that this risk stemming from parental AvPD may, in part, be mediated through parenting practices that increase the risk of a range of psychosocial outcomes in offspring, which might, in turn, increase their risk of developing AvPD traits (Wichstrøm et al., 2023; Wilson, Stroud, & Durbin, 2017).

A notable set of assumed environmental risk factors received no support, such as negative life events, a lack of close relationships with other adults (here: the primary teacher), and bullying victimization. Their status as assumed risk factors mainly stem from retrospective reports from patients. Possibly, the patients' current situations affected their recall and interpretation of past events. Although we were not able to identify environmental predictors, it should be noted that approximately half of the variation in AvPD traits is due to non‐heritable variation (Reich & Schatzberg, 2021). Thus, delineating these specific environmental factors and determining under which circumstances they make children vulnerable is a task awaiting future research.

Several basic psychological functions—executive functioning, understanding others' emotions, avoidant attachment, and assertiveness—were also not found to be predictive. A strong case for early‐formed avoidant attachment as part of the etiology of AvPD has been made (Lampe, 2016), but the support for this comes from studies of adult patients. Prospective studies starting in childhood, however, find no stability in secure attachment when the time span between assessments exceeds 15 years (Pinquart, Feussner, & Ahnert, 2013). For shorter time intervals, the stability is still modest—and weaker for those with insecure attachment. These developmental findings suggest that no relation should be expected between childhood avoidant attachment and adult AvPD, aligning with the present results. Taken together, our findings suggest that impairment in fundamental psychological functioning is not the reason why some individuals develop AvPD traits. Instead, difficulties in factors more closely related to the AvPD construct—poor self‐worth and approach–avoidance conflict in taking social initiatives—may be at play.

The 3.2% prevalence among adolescents found in this study is close to the global average of 2.7% in adults (Winsper et al., 2020). As for adults (Hummelen et al., 2006; Reich & Schatzberg, 2021), a clear one‐factor solution for AvPD emerged. Adolescence is seen as a period of renegotiation of social relationships and identity exploration, resulting in much interpersonal and intrapersonal change, making the needed stability in behavior, thinking, and emotions for the assessment of PDs difficult. Even so, studies indicate that PD traits can be reliably and validly measured in adolescence, and the stability in PD traits from adolescence to adulthood is no less than that in adulthood (d'Huart et al., 2023). The present results should therefore also have implications for AvPD traits in adulthood.

### Limitations

Although the present study had many strengths, such as involving a birth cohort sample and performing a biennial assessment of the development of risk factors from an early age with multiple sources, we acknowledge some limitations. First, although AvPD is likely dimensional in nature, we cannot be certain that the present findings also apply to the diagnosis of AvPD. Second, we only studied main effects. The risk factors in the current inquiry are likely intertwined and influence each other in complex ways. Hence, factors that were predictive at the univariable level but not in multivariable models might still convey increased risk, mediated through the covariates, social anxiety, and neuroticism. However, the number of interactions that could be theoretically argued for is vast. The sample size prevented us from testing a cross‐lagged model with all predictors, including their interactions, which could have identified mediators and moderators. Third, we measured AvPD traits only at 16 years of age. These traits might manifest even earlier, and the predictors identified in the current report might be consequences of early AvPD traits or even early manifestations of such traits. Fourth, AvPD has been found to be more prevalent in Norway than in many other countries, and our sample was primarily white and well‐educated; thus, the findings might not be generalizable to other countries and ethnicities.

## Conclusions

Although it is believed that AvPD has childhood origins, little prospective research exists to substantiate such claims. Data from this birth cohort sample measured biennially from 4 to 16 years of age show no support for the childhood origins described in the DSM‐5, as shyness and fear of new situations and people did not forecast AvPD traits. Presumed environmental risk factors were also unpredictive. However, several child‐related factors forecasted AvPD traits at 16 years of age: high and increasing levels of neuroticism and SAD symptoms, low and decreasing extraversion, declining self‐worth, high levels of onlooking behavior in childhood, and AvPD traits in parents.

## Ethical approval

The Regional Committee for Medical and Health Research Ethics approved the project, Mid‐Norway (approval number 2019/509). Health nurses informed parents about the TESS, and written consent to participate was obtained from parents. At 16 years of age, the legal consent age in Norway for medical and research purposes, written consent was obtained from the adolescents themselves.

**Key points**

- AvPD is prevalent in young adults. It is assumed to originate in childhood, starting with shyness and fear of new people and situations, but there is little prospective research to substantiate this claim.
- By following a birth cohort sample from preschool to adolescence, there was no support for avoidant attachment or early emerging shyness and fear to predict AvPD traits at 16 years.
- More AvPD traits were predicted by early neuroticism and low extraversion, more and increasing symptoms of social anxiety, declining self‐worth, increasing passive onlooking behavior in children, and higher levels of parental AvPD traits.
- Early interventions to promote social participation, increase self‐worth, and reduce social anxiety might protect against the development of AvPD later in life.

## Supporting information


**Figure S1.** Flow chart of recruitment and follow‐up.

## Data Availability

The data that support the findings of this study are not available due to consent restrictions from the participants. The analytic code supporting the findings is available upon request from the first author (LW).
