# Supplementary material for: Childhood predictors of avoidant personality disorder traits in adolescence: a seven‐wave birth cohort study
Source: J Child Psychol Psychiatry. 2024 Nov 4;66(3):366–77. doi: 10.1111/jcpp.14064 (PMC11812495; doi:10.1111/jcpp.14064)
Supplement: Supplementary file 1 — Figure S1. Flow chart of recruitment and follow‐up. [file JCPP-66-366-s001.docx]

**eFigure 1. Flow chart of recruitment and follow-up**

Invited

N=3,456

Participated T5

n=666

Did not participate T5

n=584

Participated T4

n=703

Participated T3

n=704

Did not participate T3

n=546

Asked to participate

n=3,016

Did not participate T2

n=448

Participated T2

n=802

Declined

n=539

Presented to the well-child clinic

n=3,358

Participated T7

n=665

Participated T6

n=636

n=1112

n=82

Did not participate T7

n=585

n=19

n=56

n=39

n=40

n=36

n=134

Did not participate T6

n=614

n=60

n=30

Excluded

n=176

Missed being asked to participate

n=166

Met inclusion criteria

n=3,182

Participated T1

n=1007

Drawn to participate

n=1,250

Consented

n=2,475

Did not participate T1

n=243

Did not participate T4

n=547

n=242

n=37
